# Influence of Isoflavones and Probiotics on Magnesium Status in Healthy Female Rats

**DOI:** 10.3390/foods12213908

**Published:** 2023-10-25

**Authors:** Iskandar Azmy Harahap, Maciej Kuligowski, Marcin Schmidt, Paweł Kurzawa, Joanna Suliburska

**Affiliations:** 1 Department of Human Nutrition and Dietetics, Faculty of Food Science and Nutrition, Poznan University of Life Sciences, 60-624 Poznan, Poland; iskandar.harahap@up.poznan.pl; 2 Department of Food Technology of Plant Origin, Faculty of Food Science and Nutrition, Poznan University of Life Sciences, 60-624 Poznan, Poland; maciej.kuligowski@up.poznan.pl; 3 Department of Biotechnology and Food Microbiology, Faculty of Food Science and Nutrition, Poznan University of Life Sciences, 60-624 Poznan, Poland; marcin.schmidt@up.poznan.pl; 4 Department of Clinical Pathology, Poznan University of Medical Sciences, 60-355 Poznan, Poland; pawel.kurzawa@skpp.edu.pl

**Keywords:** tempeh, isoflavones, probiotics, minerals, bone cells, healthy female

## Abstract

Isoflavones and probiotics are promising nutrients for bone health, and magnesium (Mg) is essential for bone metabolism. This study aimed to determine the effects of daidzein, genistein and *Lactobacillus acidophilus* on the Mg status of healthy female rats. Forty-eight rats were randomly assigned to six groups, with the control group receiving a standard diet (AIN 93M). The remaining groups were fed the same diet with added ingredients such as tempeh flour; soy flour; pure daidzein and genistein; *L. acidophilus* or a combination of daidzein, genistein, and *L. acidophilus*. Tissue samples were collected after the eight-week intervention, and Mg concentrations were analysed using flame atomic absorption spectrometry. Myeloid and erythroid cells were determined using the haematoxylin and eosin bone staining method. Statistical analyses were conducted using one-way ANOVA with Tukey’s post hoc test and Pearson’s correlation coefficient. The threshold for significance was *p* < 0.05. Compared with the control group, adding tempeh to the diet of rats resulted in significant changes in Mg concentrations in various tissues, with a decrease in the kidneys and an increase in the fur. Although not statistically significant compared to the control group, the tempeh group showed increased Mg concentrations in the femur and spleen. The myeloid-to-erythroid cell ratio did not differ significantly among groups, but all intervention groups showed higher ratios than the control group. A strong negative correlation was observed between Mg concentrations in the kidneys and fur. Conversely, a positive correlation was identified between Mg concentrations in the pancreas and fur. Daily consumption of tempeh may improve Mg status in the organism. Intake of pure daidzein, genistein, or probiotic seems to have no effect on Mg concentrations in healthy rats.

## 1. Introduction

Magnesium (Mg) is an essential mineral that plays a crucial role in the adequate functioning of many physiological processes in maintaining human bone health [1]. Postmenopausal women are a population that is particularly susceptible to Mg deficiency [2]. Various factors influence the body’s Mg status, including dietary intake [3], absorption [4], hormonal changes [5], and certain medications [6]. Other factors such as age, gender, and health status can also affect Mg status [7]. For instance, Mg absorption decreases with age, and certain health conditions, such as gastrointestinal disorders, can affect Mg absorption and excretion [8]. Certain medications, such as diuretics and proton pump inhibitors, can also affect Mg status by increasing urinary excretion or reducing intestinal absorption [9]. Furthermore, the decreased oestrogen levels found in postmenopausal women are associated with reduced Mg absorption and increased excretion, leading to an increased risk of Mg deficiency [10]. Thus, understanding the factors that influence Mg status is crucial in preventing Mg deficiency and maintaining bone health.

Bone health can be negatively impacted by Mg deficiency in multiple ways. Low Mg concentrations affect the apatite crystal structure. Compared to healthy women, women with osteoporosis who have been shown to have a Mg deficiency have more ordered crystals in their trabecular bone [11]. Histologically, anaemia commonly causes an increase in erythropoietic cells (erythroid hyperplasia), which can be seen on a tissue section under microscopy (e.g., early blood loss or haemolytic anaemia). Nevertheless, inflammation is frequently linked to increased granulopoietic cells (myeloid hyperplasia). Morphologically, this could mean an increase in the myeloid-to-erythroid cell ratio and a reordering of the maturation process that occurs later in the cell’s life cycle [12]. Moreover, a previous study reported that Mg deficiency is associated with an increased risk of anaemia, particularly in women and older adults [13].

In postmenopausal women with osteopenia, the combined use of isoflavones and probiotics has been shown to improve bone health and oestrogen metabolism. Supplementation with calcium, Mg, and calcitriol proved more beneficial when paired with a diet high in isoflavones and probiotics [14]. Meanwhile, our earlier research showed that healthy male rats with increased consumption of multispecies probiotics had a much higher Mg concentration in their femurs [15].

Probiotics influence calcium absorption by modulating transcellular and paracellular pathways within the intestinal epithelium, promoting a balanced gut microbiota, and regulating bone health via immune system modulation. Simultaneously, isoflavones facilitate calcium mobilization from bone into circulation, contributing to systemic calcium homeostasis [16]. Previous research has shown that specific Lactobacillus strains derived from dairy products enhance total calcium transport and impact the paracellular calcium absorption pathway by upregulating the cld-2 gene. Additionally, they improve intestinal calcium uptake through the transcellular pathway, involving vitamin D receptor (VDR) and transient receptor potential cation channel subfamily V member 6 (TRPV6) [17]. Furthermore, daily isoflavone intake has been associated with increased whole-body bone mineral density, particularly in healthy premenopausal women with higher serum calcium levels [18].

Regarding calcium [19,20] and iron [21] status, the effects of isoflavones and *Lactobacillus acidophilus* have been studied extensively. However, the effects of isoflavones and *L. acidophilus* on Mg status have not been studied in detail. Therefore, this study aims to determine the impact of isoflavones and *L. acidophilus* on Mg status in healthy female rats. Specifically, the study investigates the effects of daidzein, genistein, and *L. acidophilus* on the Mg distribution in tissues and the myeloid-to-erythroid cell ratio in the bone femur. The novelty of this study lies in its investigation of the effects of isoflavones and *L. acidophilus* on Mg status, which has not been thoroughly explored before.

## 2. Materials and Methods

### 2.1. Materials

The AIN 93M diet, manufactured by Zoolab in Sędziszów, Poland, was utilised as the standard rat diet, with ingredients such as Augusta variety soybeans obtained from the Department of Genetics and Plant Breeding at the Poznań University of Life Sciences, Poland, as well as pure daidzein and genistein supplied by LC Laboratories based in Woburn, MA, USA. Additionally, tempeh, which is fermented soybean made with *Rhizopus oligosporus* NRRL 2710, and probiotics in the form of *Lactobacillus acidophilus* DSM20079 were prepared according to the procedure outlined in a prior study [22]. The *R. oligosporus* NRRL 2710 used in the fermentation process was obtained from the Agricultural Research Service Culture Collection in Peoria, IL, USA, and the *L. acidophilus* DSM20079 used for the probiotics was obtained from the German Collection of Microorganisms and Cell Cultures, the Leibniz Institute DSMZ, Braunschweig, Germany.

### 2.2. Ethics of Animal Research

This study was approved by a local ethics committee in Poznań, Poland, following the National Institutes of Health’s Handbook for the Care and Use of Laboratory Animals (NIH Publication No. 80-23, Revised 1978), the Directive 2010/63/EU of the European Parliament, the Council of 22 September 2010 on the Protection of Animals Used for Scientific Purposes, and Polish law. According to the Animal Research: Reporting of In Vivo Experiments (ARRIVE) guidelines, animal studies were conducted under the guidelines established by the Poznań University of Life Sciences, Poland.

### 2.3. Conditioning the Animal Laboratory Environment

**Figure 1:**
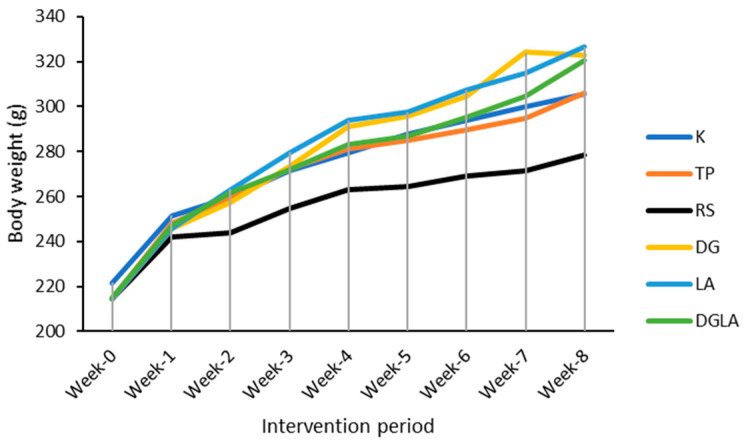
The trendline of average body weight of the rats during the study period. K: a group received the standard diet (AIN 93M); TP: a group received AIN 93M with tempeh flour; RS: a group received AIN 93M with soy; DG: a group received AIN 93M with pure daidzein and genistein; LA: a group received AIN 93M with *Lactobacillus acidophilus*; DGLA: a group received AIN 93M with daidzein, genistein, and *L. acidophilus*.

The Animal Laboratory of the Poznań University of Life Sciences, Poland, provided a stable and regulated environment for female Wistar rats. The rats were kept in a room with relative humidity ranging from 55% to 65%, a constant temperature of 21 ± 2 °C, and a light and dark cycle of 12 h each. During the adaptation and intervention phases, they were housed in pairs in stainless steel cages coated with enamel, free of any metal. The rats were given five days to acclimatise to the controlled laboratory environment. Rats had unrestricted access to Labofeed B (Żurawia, Kcynia, Poland) and tap water throughout the acclimation period. Labofeed B standard is designed as a maintenance diet for adult rats and was formulated under the recommendations of the National Research Council of Poland for Nutritional Requirements of Laboratory Animals. Figure 1 displays the average body weight (in grams) of the studied rats.

**Figure 2:**
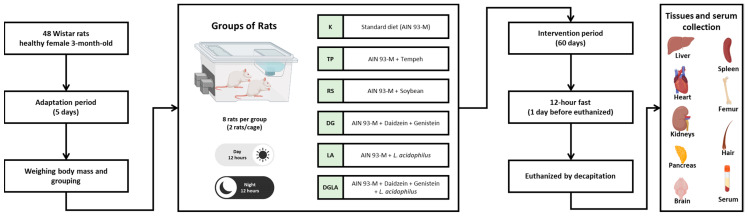
Experimental design of adaptation, grouping, intervention, and sample collection. The black arrow outlines the step-by-step flow process followed in the experiment.

Moreover, the stress experienced by the rats during the experiment was kept to a minimum by, among other means, decreasing the number of employees performing animal-related experimental procedures or reducing the frequency and duration of personnel-animal interactions. Throughout the study, the animals were under veterinary care. A flowchart that illustrates the research process is presented in Figure 2.

### 2.4. Grouping the Rats

After the adaptation phase, the rats were weighed using a calibrated scale to obtain their baseline body weights. The weight of the animals is an essential factor in the proper randomisation of experimental groups, as it ensures that each group has a similar starting point. The rats were then randomly assigned to six groups, each containing eight rats, based on their body weight. Randomisation of animals is a crucial aspect of experimental design as it reduces the risk of bias and ensures that the groups are comparable at the start of the study [23]. Previous studies have demonstrated that research involving a minimum of eight rats in each group has sufficient statistical power to identify a significant intervention effect [24].

### 2.5. Diet Intervention Groups

The control (K) group was fed AIN 93M, the tempeh (TP) group received AIN 93M with tempeh flour (250 g/kg of standard diet), and the soy (RS) group received AIN 93M with soy (250 g/kg of standard diet). The pure daidzein and genistein (DG) group was provided AIN 93M supplemented with daidzein (10 mg/kg of standard diet) and genistein (100 mg/kg of standard diet). The probiotics (LA) group was provided AIN 93M supplemented with *L. acidophilus* DSM079 (10^10^ CFU/day). The daidzein, genistein, and probiotics (DGLA) group received AIN 93M with daidzein (10 mg/kg of standard diet), genistein (100 mg/kg of standard diet), and *L. acidophilus* DSM079 (10^10^ CFU/day).

We chose the dosages of isoflavones and probiotics based on past research demonstrating their positive effects on rodents and human bone health. According to an examination of aglycon content (daidzein, glycitein, and genistein), the recommended daily intake of tempeh is 250 g [25]. To guarantee uniformity, the amount of pure daidzein and genistein was standardised to the level found in 250 g of tempeh in this study. For probiotics, we consulted a study by Dar et al. in which ovariectomised female mice were administered *L. acidophilus*-containing diets (10^9^ CFU/day) for six weeks [26]. The authors noticed a decrease in osteoclastogenic factor expression and an increase in anti-osteoclastogenic factor expression with a daily dose of 10^9^ CFU/day. However, the effect of the high dose of probiotics (1 × 10^10^ CFU/day) was more pronounced in postmenopausal women than that of the low dose (2.5 × 10^9^ CFU/day) [27]. Hence, we selected a 10^10^ CFU/day dose of *L. acidophilus* for this study. In addition, the composition of the standard diet and other modified diets are presented in Table 1.

### 2.6. Monitoring the Diets and the Rats during the Intervention Period

During the experimental phase, the rats in each group were weighed on a weekly basis using the same calibrated scale as during the adaptation phase. The daily food intake of each group was also recorded throughout the experimental phase. To ensure that the rats had unlimited access to the diet and tap water, they were provided with fresh food and water each day, while any leftover food and water from the previous day was removed. This standard protocol ensured that the rats received consistent food and water, which is essential for maintaining their health and normal growth [28]. Any changes in their weight or food intake could indicate potential health issues, and thus monitoring these variables is critical to conducting a reliable and valid experiment. Additionally, by providing fresh food and water each day and removing the leftovers, the researchers were able to prevent spoilage and ensure that the rats were consuming fresh and uncontaminated food and water throughout the experiment.

### 2.7. Decapitating the Rats

After the eight-week intervention period, the rats were subjected to a 12-h fasting period before body weight measurement. This fasting period was initiated to minimise the potential impact of the rats’ recent food intake on subsequent measurements. Once the rats were weighed, they were decapitated, and their heads were immediately removed. Serial sections were then made of the whole body to obtain the necessary tissue samples for analysis. It is important to note that decapitation is a widely accepted and ethical method of euthanasia in laboratory animals, as it causes rapid and painless death. The tissue samples obtained from the rats were subsequently used for further analysis, such as histological or biochemical assays, to investigate the effects of the intervention on the targeted physiological processes.

### 2.8. Collection of Tissues and Serum

After decapitation of the rats, a total blood sample was collected via cardiac puncture. The blood was collected using a sterile syringe and transferred to a sterile tube. Dissected tissues were then cleaned in saline to remove excess blood and weighed using a precision scale. The tissues included the liver, heart, kidneys, spleen, pancreas, femur, and brain. Afterwards, the tissues were immediately frozen at a temperature of −80 °C to prevent any degradation of the samples. In addition to the tissues, fur samples were also collected from the rats. The fur samples were shaved from the same interscapular region on all rats. The fur samples were collected to examine any potential changes in fur growth or texture, which could indicate changes in the rats’ overall health status. The fur samples were then stored in a sterile container until analysis.

### 2.9. Determination of Mg Concentration in Diets and Tissues

To determine the Mg contents of the diets, 2 g of each diet was weighed and ashed in a muffle furnace at 450 °C until complete mineralisation. The resulting ash was dissolved in 1N nitric acid (Suprapure, Merck, Kenilworth, NJ, USA), and Mg concentrations were determined using flame atomic absorption spectrometry (AAS-3, Carl Zeiss, Jena, Germany), following the method of Suliburska et al. [15].

To determine the Mg contents in the various tissues (liver, heart, kidneys, spleen, pancreas, femur, brain, and fur), they were first digested in 65% (*w*/*w*) spectra pure HNO_3_ (Merck, Kenilworth, NJ, USA) using a Microwave Digestion system (Speedwave Xpert, Berghof, Eningen, Germany), following the procedure of Suliburska et al. [15]. After digestion and subsequent dilution with deionised water, the Mg contents of the mineral solutions were measured using flame atomic absorption spectrometry (AAS-3, Carl Zeiss, Jena, Germany).

The Mg contents of both the diets and the tissues were measured at a wavelength of 285.2 nm. The accuracy and reliability of the method were confirmed using certified reference materials. A certified reference material of soybean flour INCT-SBF-4 (Institute of Nuclear Chemistry and Technology, Warszawa, Poland) was used to determine the Mg contents of diets with 98% accuracy, while a certified reference material of bovine liver NIST-1577C (Sigma-Aldrich, Saint Louis, MO, USA) was used to determine tissue Mg contents with 91% accuracy.

### 2.10. Haematoxylin and Eosin (H & E) Staining of Bone

After surgery, femoral bones were collected as specimens and fixed in 10% buffered formalin for 24 h. Subsequently, the specimens were immersed in a decalcifying solution, namely the Osteodec bone marrow biopsy decalcifying solution, for 3 h. The specimens were then processed and individually embedded in paraffin blocks according to the standard operating procedure. Three 2 µm slices were obtained from each paraffin block and stained with haematoxylin and eosin (H & E). Two femoral bone slices with their bone marrow content were present on each slide. Using a light microscope (Leica, Allendale, NJ, USA), two researchers independently counted the number of myeloid and erythroid cells in each sample in a high-power field. The myeloid and erythroid counts were recorded and analysed for statistical significance.

### 2.11. Statistical Analysis

The normality of the variable distributions was assessed using the Shapiro–Wilk test. Statistical significance was established using the Tukey post hoc test following analysis of variance (ANOVA). Disparities were considered statistically significant at a probability level of 5% (*p* < 0.05). Pearson’s correlation was utilised to examine the correlation between Mg concentrations in serum and tissues. The Mg concentration in diets was measured in triplicate, and the data were reported as mean and standard deviation.

## 3. Results

### 3.1. Body Weight Gain and Mg Intake

The results showed significant changes in body weight gain among the different groups. Specifically, the soybean (RS) group exhibited a 30% significant decrease in body weight gain, while the *L. acidophilus* (LA) group, the pure daidzein and genistein (DG) group, and the combination of daidzein, genistein, and *L. acidophilus* (DGLA) group showed a significant increase in body weight gain by 40%, 36%, and 32%, respectively, compared to the control group after eight weeks of consumption, as shown in Table 2.

The food intake among the different groups also showed significant changes during the eight-week intervention. Specifically, the tempeh (TP) and soybean (RS) groups significantly decreased food intake by 4% and 9%, respectively, compared to the control group. By contrast, the pure daidzein and genistein (DG), *L. acidophilus* (LA), and their combination (DGLA) groups all showed significant increases in food intake by 17%, 14%, and 13%, respectively, compared to the control group.

In terms of the Mg content in the diet, the tempeh (TP) and soybean (RS) groups had the highest Mg content, which was significantly higher than that of the control group. Conversely, the *L. acidophilus* (LA) group had the lowest Mg content in the diet, which was also significantly lower than that of the control group. All intervention groups had significantly higher Mg intake than the control group, but the tempeh (TP) group had the highest Mg intake.

### 3.2. Mg Concentrations in Serum and Tissues

In the present study, the Mg concentrations in various tissues and serum were determined in different groups of rats. As shown in Table 3, the Mg concentration in the kidneys decreased significantly by 7% in the tempeh (TP) group compared to the control group. By contrast, the tempeh (TP) group also exhibited a significant increase in the Mg concentration in fur by 67% compared to the control group.

Although not statistically significant compared to the control group, the tempeh (TP) group had the highest Mg concentrations in the femur, while the combination of daidzein, genistein, and *L. acidophilus* (DGLA) had the lowest Mg concentration in the femur. The Mg concentration in the spleen increased by 7% in the tempeh (TP) group, and the tempeh group (TP) had the highest Mg concentration of all groups. In the pancreas, while the soybean (RS) group exhibited a significantly higher concentration of Mg and the pure daidzein and genistein (DG) group showed a significantly lower concentration of Mg, these alterations did not differ significantly from the control group. Furthermore, the Mg concentration in serum, liver, heart, and brain did not change significantly among all groups.

### 3.3. Myeloid-to-Erythroid Cell Ratio in Femoral Bone

**Figure 3:**
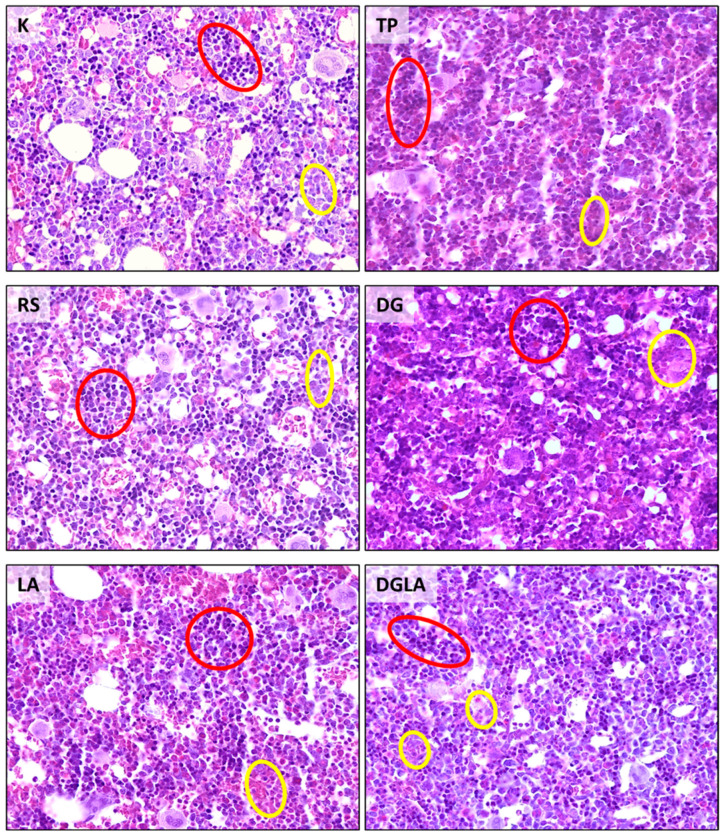
Microscopic comparison of myeloid and erythroid series in the femoral bone marrow using 400× magnification. The red circle highlights the myeloid series, while the yellow circle represents the erythroid series. K: a group received the standard diet (AIN 93M); TP: a group received AIN 93M with tempeh flour; RS: a group received AIN 93M with soy; DG: a group received AIN 93M with pure daidzein and genistein; LA: a group received AIN 93M with *Lactobacillus acidophilus*; DGLA: a group received AIN 93M with daidzein, genistein, and *L. acidophilus*.

In this study, although no statistically significant changes were observed in the myeloid and erythroid series of all groups, the soybean (RS) group had the highest myeloid-to-erythroid cell ratio of all the groups (Table 4). However, all intervention groups resulted in an increased myeloid-to-erythroid cell ratio compared to the control group. Figure 3 displays the visual representation of the myeloid and erythroid series in the femoral bone of all experimental groups.

### 3.4. Correlation between Mg Concentration in Serum and Tissues

Significant correlations between Mg concentrations in serum and tissues, such as the liver, heart, kidneys, spleen, pancreas, femur, brain, and fur, are shown in Table 5. The strongest negative correlation was found between the kidney Mg concentration and the fur Mg concentration (−0.280). Meanwhile, the strongest positive correlation was seen between the pancreatic Mg concentration and the fur Mg concentration (0.548).

## 4. Discussion

Our findings revealed several notable results. The tempeh group exhibited the highest Mg concentrations in the femur, spleen, and fur. This finding suggests that tempeh consumption increases Mg intake and may also contribute to an increase in its bioavailability. The effect of tempeh on the distribution of Mg in the body was also visible. Watanabe et al. also reported the result of consuming fermented soybeans on the Mg concentrations in the femurs of rats. The diet intervention was given to the rats for four weeks. Even though no statistically significant differences were discovered, consuming tempeh resulted in a higher Mg concentration in the femur (0.19%) compared to consuming soybeans (0.17%) [29].

Higher Mg concentrations in bones benefit bone health, improving bone mineral density, which can help prevent bone diseases such as osteoporosis [30]. Mg is involved in various cellular processes and plays a vital role in bone metabolism, modulating the activity of osteoblasts and osteoclasts [31]. However, in this study, we used healthy rats, and the increase in Mg in the bones over a longer period of time could have had a negative effect on osseous metabolism. Investigating this process will require longer studies. According to a previous study, postmenopausal women with the highest Mg intake had the highest incidence of wrist fractures [32].

Compared with the soybean and pure isoflavone groups, the tempeh group showed a significant increase in Mg concentration in the spleen. This result suggests that tempeh consumption may uniquely affect Mg storage or utilisation in this particular organ. The spleen plays a vital role in maintaining peripheral tolerance by clearing apoptotic cells, thereby regulating the immune system [33]. Specialised T-cells and B-cells are present within the spleen’s white pulp, which are responsible for generating new immune cells and antibodies [34,35]. Additionally, the spleen serves as a reservoir for circulating monocytes, facilitating their rapid recruitment to sites of inflammation [36,37,38]. Our recent work found that tempeh altered neutrophil and lymphocyte counts in the blood of healthy female rats [21], suggesting that tempeh may have anti-inflammatory properties.

Interestingly, the tempeh group also exhibited a significant increase in the Mg concentration in fur compared to the control group. In a cross-sectional study conducted in Korea, 104 premenopausal volunteers from outpatient clinics and a health promotion centre at a university hospital were assessed to investigate the relationship between fur minerals and bone mineral density in premenopausal women. The findings revealed a significant association between Mg concentrations in fur and bone mineral density in premenopausal women [39]. The findings of this study suggest that tempeh consumption may influence the absorption and distribution of Mg in the body, leading to the changes in Mg concentrations observed in specific tissues.

Our study also revealed significant differences in Mg concentrations in other organs. Possible explanations for the decrease in Mg levels in the kidneys could involve altered renal handling due to specific components present in tempeh. It is known that the kidneys are sensitive regulators of Mg status [38]. The higher Mg concentrations in the fur, spleen, and femur with decreased Mg concentrations in the kidney could be due to the effective absorption and distribution and accumulation of Mg facilitated by tempeh.

This result demonstrates that a high source of Mg from tempeh may have resulted in more Mg being available for distribution to the bones (femur), spleen, and fur, which may lead to decreased extraction of Mg through urine. The higher Mg concentrations in the fur of the tempeh group may be indicative of Mg status in the organism, potentially leading to Mg loss during the natural fur growth cycle. This finding highlights the importance of considering the overall impact of dietary interventions on Mg status, taking into consideration the bioavailability, distribution, and renal excretion.

The noteworthy observation of more pronounced differences in Mg status, especially concerning dietary variations, underscores the potential of fur as a valuable indicator of physiological changes, particularly in mineral status. Fur, serving as an external biomarker, can effectively mirror shifts in mineral homeostasis due to its connection with nutrient absorption and utilisation [40,41,42]. The integumentary system, inclusive of fur, has long been acknowledged for its sensitivity to changes in mineral levels within the body [43].

For instance, in a prior experimental study, it was evident that fur’s elemental composition changed in tandem with shifts in the elemental profile within the muscular tissue of Wistar rats exposed to modified diets for a 60-day period [44]. Another investigation revealed that Wistar Hannover rats exhibited noticeable fur-related changes after just two weeks of consuming high-fat diets. These changes were characterized by the development of a shiny and rigid coat, alongside instances of fur loss [45]. Furthermore, in human studies, hair has been recognized as a long-term biological tissue indicator for assessing the nutritional status of both men and women [46]. These findings underscore the sensitivity of fur composition as an indicator of underlying physiological changes influenced by dietary modifications.

In our current study, apart from the significant increase observed in fur, daily tempeh intake over 8 weeks induced alterations in other tissues, although these changes did not reach statistical significance compared to the control group. Notably, the tempeh group exhibited increased Mg levels in the femur, spleen, and pancreas (Table 3). These findings collectively suggest that tempeh may contribute to the improvement of Mg status within the organism.

These observations underscore the potential utility of fur as a non-invasive, easily accessible marker for assessing mineral status, contributing to a more comprehensive understanding of the complex interplay between dietary components and their impact on physiological responses in the studied organism. Further investigations into the mechanisms underpinning fur-related findings may illuminate the intricacies of mineral metabolism and its influence on external biomarkers.

The complexity of regulating Mg status in the body was reflected in the correlations we observed in our study. A negative correlation was observed between kidney and fur Mg concentrations. This result indicates an inverse relationship between the concentrations of Mg in the kidneys and the concentrations of Mg deposited in the fur. Thus, the findings suggest a change in Mg distribution in rats on a tempeh diet, leading to a lower kidney Mg concentration and higher Mg concentrations in the fur.

The dietary composition in our study indeed plays a pivotal role in influencing Mg levels and absorption, a subject that warrants detailed examination. The intricate relationship between macronutrients and Mg balance is crucial, with macronutrients being key players in the regulation of nutritional absorption [47]. They undergo digestion and absorption processes, potentially instigating significant physiological responses [48].

Specifically, soybean and tempeh are characterized by significantly high levels of protein, fat, fibre, and carbohydrates compared to the other diet groups (Table 1). A consistent decline in both the absolute and relative measurable Mg absorption was observed in rats fed diets containing soybean protein [49]. These dietary components hold the potential to impact Mg bioavailability. Mg absorption is influenced by various factors. Certain factors, such as partly fermentable fibres like hemicellulose, non-fermentable fibres like cellulose and lignin, phytate, and oxalate, tend to impair Mg absorption. Conversely, proteins, medium-chain triglycerides, and low- or indigestible carbohydrates like resistant starch, oligosaccharides, inulin, mannitol, and lactulose have been shown to enhance Mg uptake [8]. These factors collectively contribute to the intricate regulation of Mg absorption within the body.

In the context of our present study, it is essential to consider that the effects on Mg absorption may not be as pronounced due to our use of healthy rats. This is an important factor to highlight in our discussion, emphasizing that the overall impact on Mg status might be relatively modest compared to studies involving subjects with compromised health conditions.

Despite the known positive physiological effect of probiotics on Mg availability [50], Mg status [15], and Mg retention [51], our current study did not observe this effect when *L. acidophilus* was added to the diet. Our results agree with those of previous studies [52]. Moreover, one possibility is that the probiotic strain used in the study may not have been effective at enhancing Mg absorption or utilisation. Another possibility is that the dose or duration of probiotic supplementation was not optimal to induce a significant effect. Other factors, such as individual variations in gut microbiota composition, genetic predispositions, or dietary factors, could have also influenced the study’s outcomes. Thus, further research is needed to elucidate the underlying mechanisms and determine the optimal conditions for probiotics to affect Mg metabolism.

This study found that consumption of *L. acidophilus* led to a significant increase in body weight gain compared to the control group. This increase in weight gain may have been due to several causes. Firstly, it is possible that the bacteria improved the absorption of macronutrients [53], increasing weight gain. Secondly, the bacteria may have altered the gut microbiome [54], which can affect metabolism and contribute to weight gain. Lastly, it is possible that the increase in weight gain was due to increased food intake, as some studies have suggested that *L. acidophilus* can stimulate appetite [55].

Histological findings of this study demonstrated that the observed increase in the myeloid-to-erythroid cell ratio in the intervention groups compared to the control group indicates a shift in haematopoiesis towards increased myeloid cell production and decreased erythroid cell production. This alteration may have implications for the immune response, inflammation, and bone health [56]. Although the liver and spleen have been found to have haematopoietic capabilities, the bone marrow continues to be the major haematopoietic and lymphoid organ in most adult animals responsible for erythropoiesis, myelopoiesis, and thrombopoiesis [57]. We confirm a prior finding that the observational group had a greater myeloid-to-erythroid cell ratio than rats in the control group [58]. In addition, it has been shown in animal models that Mg deficiency causes chronic inflammation. Low Mg accelerates bone loss because of the inflammatory cytokines that are produced, particularly TNF-α and IL-1 [59]. The results of this study indicate that the increased myeloid-to-erythroid cell ratio in isoflavones and probiotic dietary products may contribute to an improvement in the immune response and inflammation, which may have an effect on Mg status in bone health.

Our study conducts a comprehensive investigation into the impact of dietary components on Mg status in healthy female rats. By comparing our findings with existing research, we aim to highlight the distinct advantages of our results. Notably, a prior study reported that isoflavone extract, when coupled with calcium, Mg, and calcitriol supplementation, exhibited enhanced effectiveness compared to supplementation alone in postmenopausal osteopenic women [14]. In alignment with these findings, our research underscores the potential therapeutic role of tempeh, a soy-based food known for its high isoflavone content, in significantly improving Mg status within the organism. This observation distinguishes our study and emphasizes the potential of tempeh as a dietary intervention for maintaining Mg homeostasis. Through this comparative analysis, we shed light on the unique and valuable insights gained from our research, thereby amplifying the significance of our study within the realm of Mg metabolism.

Several limitations should be considered when interpreting the findings of this study. Firstly, the study was conducted exclusively on female rats, which may limit the generalizability of the results to other populations, including male rats. Secondly, using healthy rats may not fully capture the potential effects of isoflavones and probiotics in individuals with underlying health conditions, particularly osteoporotic postmenopausal women. Another limitation is that this study did not measure specific blood biomarkers related to Mg metabolism. Assessment of biomarkers, such as Mg transporters or inflammatory markers, could have shed light on the mechanisms underlying Mg distribution.

In addition to these limitations, the process of Mg absorption is a multifaceted physiological mechanism that plays a crucial role in maintaining the Mg levels within an organism [60]. The analysis of faecal Mg concentration can offer significant insights into the degree of absorption and utilisation of ingested Mg within the gastrointestinal system [61]. This form of analysis has the potential to provide insights on the effectiveness of Mg absorption, thereby providing a holistic understanding of Mg homeostasis. Therefore, this study possesses the potential to serve as a promising path for more exploration, highlighting its significance for future research endeavours.

Considering these limitations, further research is warranted to explore the clinical impact of the findings in a nutritional context. Future studies could focus on conducting randomised controlled trials involving human subjects to investigate the effects of isoflavones and probiotic supplementation on Mg status and associated health outcomes.

Such studies could evaluate the effects of isoflavone consumption or probiotic supplementation on specific clinical markers, including a comprehensive assessment of Mg transporters, inflammatory markers, and other relevant biochemical pathways that could provide a deeper understanding of the mechanisms involved in the observed effects. Additionally, investigating potential interactions between tempeh and other dietary components and considering individual variations in nutrient metabolism would provide valuable insights for personalised nutrition approaches.

Overall, future research should aim to elucidate the clinical implications of isoflavones and probiotics in diverse populations, address potential confounding factors, and explore the underlying mechanisms to guide evidence-based dietary recommendations and improve nutritional interventions for optimising Mg status and promoting bone health.

## 5. Conclusions

In summary, our study provides valuable insights into the effects of dietary components on Mg status in healthy female rats. The results suggest that daily consumption of tempeh, a soy-based food rich in isoflavones, may enhance Mg status within the organism. This finding underscores the potential beneficial role of tempeh in maintaining Mg homeostasis. Contrastingly, our investigation revealed that the intake of pure daidzein, genistein, or probiotics, while considered important components, did not exhibit a discernible impact on Mg concentrations in healthy rats. Tempeh exhibits a whole matrix, encompassing elements such as Mg, macronutrients, and isoflavones. The interactions among these components may explain the absence of a significant impact from pure isoflavones. However, further research is warranted to delve deeper into the mechanisms underlying these effects and to explore potential implications for human nutrition and health. This study lays the foundation for future investigations aimed at elucidating the intricate interplay between dietary components and Mg homeostasis.

## Figures and Tables

**Table 1:** Nutritional content of each type of feed (adapted with permission from Harahap et al. [22]. 2022, Acta Scientiarum Polonorum Technologia Alimentaria).

Group	Protein (g/100 g)	Fat (g/100 g)	Insoluble Fibre (g/100 g)	Soluble Fibre (g/100 g)
K	14.95 ± 0.64 ^a^	4.28 ± 0.19 ^a^	5.92 ± 0.45 ^a^	1.87 ± 0.03 ^a^
TP	23.56 ± 0.83 ^c^	7.61 ± 0.23 ^c^	11.61 ± 0.75 ^b^	3.78 ± 0.04 ^c^
RS	21.04 ± 0.89 ^b^	5.14 ± 0.02 ^b^	14.88 ± 0.66 ^c^	2.76 ± 0.31 ^b^
DG	14.11 ± 0.14 ^a^	4.51 ± 0.05 ^a^	5.67 ± 0.01 ^a^	1.92 ± 0.05 ^a^
LA	14.19 ± 0.17 ^a^	4.46 ± 0.17 ^a^	5.71 ± 0.02 ^a^	1.83 ± 0.03 ^a^
DGLA	14.16 ± 0.34 ^a^	4.61 ± 0.08 ^a^	5.98 ± 0.13 ^a^	1.86 ± 0.08 ^a^
**Group**	**Fibre** **(g/100 g)**	**Carbohydrates** **(g/100 g)**	**Ash** **(g/100 g)**	**Energy** **(Kcal/100 g)**
K	7.78 ± 0.42 ^a^	70.78 ± 0.62 ^c^	2.68 ± 0.07 ^a,b^	346.45 ± 1.83 ^c^
TP	15.38 ± 0.78 ^b^	59.37 ± 1.20 ^a^	2.92 ± 0.09 ^b^	337.89 ± 3.18 ^b^
RS	17.64 ± 0.96 ^c^	63.13 ± 0.97 ^b^	3.26 ± 0.12 ^c^	311.49 ± 3.37 ^a^
DG	7.58 ± 0.04 ^a^	71.28 ± 0.20 ^c^	2.45 ± 0.08 ^a^	351.83 ± 0.57 ^c^
LA	7.54 ± 0.01 ^a^	71.34 ± 0.61 ^c^	2.51 ± 0.15 ^a^	352.14 ± 0.72 ^c^
DGLA	7.83 ± 0.07 ^a^	71.07 ± 0.32 ^c^	2.47 ± 0.04 ^a^	351.17 ± 0.68 ^c^

Data are presented as mean ± standard deviation. K: a group received the standard diet (AIN 93M); TP: a group received AIN 93M with tempeh flour; RS: a group received AIN 93M with soy; DG: a group received AIN 93M with pure daidzein and genistein; LA: a group received AIN 93M with *Lactobacillus acidophilus*; DGLA: a group received AIN 93M with daidzein, genistein, and *L. acidophilus*. ANOVA was performed using Tukey’s test. ^a–c^ represent significantly different mean values ± SD at *p* < 0.05.

**Table 2:** Body weight gain, Mg content in the diet, food intake, and Mg intake.

Group	Body Weight Gain (%)	Mg Content in Diet (g/kg)	Food Intake (g/day)	Mg Intake (μmol/g/day)
K	32.55 ± 6.64 ^b^	0.60 ± 0.02 ^b^	19.14 ± 2.63 ^c^	0.47 ± 0.06 ^a^
TP	36.13 ± 3.47 ^b,c^	1.05 ± 0.01 ^c^	18.43 ± 2.40 ^b^	0.79 ± 0.10 ^e^
RS	22.91 ± 2.96 ^a^	1.00 ± 0.02 ^c^	17.46 ± 3.10 ^a^	0.72 ± 0.13 ^d^
DG	44.30 ± 9.17 ^c,d^	0.57 ± 0.01 ^a,b^	22.32 ± 2.64 ^d^	0.52 ± 0.06 ^c^
LA	45.42 ± 4.12 ^d^	0.53 ± 0.02 ^a^	21.82 ± 2.51 ^d^	0.48 ± 0.05 ^a,b^
DGLA	42.87 ± 5.30 ^c,d^	0.56 ± 0.03 ^a,b^	21.68 ± 2.35 ^d^	0.50 ± 0.05 ^b,c^

Data are presented as mean ± standard deviation. K: a group received the standard diet (AIN 93M); TP: a group received AIN 93M with tempeh flour; RS: a group received AIN 93M with soy; DG: a group received AIN 93M with pure daidzein and genistein; LA: a group received AIN 93M with *Lactobacillus acidophilus*; DGLA: a group received AIN 93M with daidzein, genistein, and *L. acidophilus*. ANOVA was performed using Tukey’s test. ^a–e^ represents significantly different at *p* < 0.05.

**Table 3:** Mg concentration in serum and tissues.

Group	Serum (μmol/mL)	Liver (μmol/g)		Femur (μmol/g)		Kidney (μmol/g)		Brain (μmol/g)
K	1.02 ± 0.09	30.94 ± 3.09		163.10 ± 11.47 ^a,b^		31.99 ± 0.91 ^b^		23.87 ± 1.62
TP	0.98 ± 0.03	29.94 ± 1.87		172.49 ± 10.39 ^b^		29.89 ± 1.66 ^a^		24.06 ± 0.53
RS	0.98 ± 0.06	34.91 ± 6.86		164.64 ± 8.01 ^a,b^		30.61 ± 1.94 ^a,b^		24.04 ± 0.90
DG	1.04 ± 0.03	30.50 ± 2.08		165.16 ± 7.82 ^a,b^		31.46 ± 0.84 ^a,b^		24.95 ± 0.57
LA	1.03 ± 0.06	31.52 ± 1.48		160.85 ± 5.41 ^a,b^		31.91 ± 1.45 ^a,b^		26.01 ± 2.69
DGLA	1.05 ± 0.12	34.31 ± 4.70		155.45 ± 7.91 ^a^		30.78 ± 0.94 ^a,b^		24.39 ± 0.82
**Group**	**Heart (μmol/g)**		**Spleen (μmol/g)**		**Pancreas (μmol/g)**		**Hair (μmol/g)**	
K	34.21 ± 1.76		38.75 ± 3.41 ^a,b^		36.49 ± 2.56 ^a,b,c^		4.84 ± 0.69 ^a,b^	
TP	34.34 ± 1.20		41.49 ± 3.31 ^b^		38.40 ± 3.94 ^b,c^		8.10 ± 1.14 ^c^	
RS	34.06 ± 0.68		36.62 ± 1.31 ^a^		40.85 ± 1.92 ^c^		5.92 ± 0.74 ^b^	
DG	35.63 ± 1.82		36.23 ± 1.69 ^a^		32.54 ± 1.98 ^a^		4.70 ± 0.61 ^a^	
LA	34.69 ± 2.05		38.24 ± 3.18 ^a,b^		36.09 ± 3.61 ^a,b^		5.09 ± 0.31 ^a,b^	
DGLA	34.99 ± 2.08		38.89 ± 2.45 ^a,b^		34.76 ± 3.44 ^a,b^		4.40 ± 0.66 ^a^	

Data are presented as mean ± standard deviation. K: a group received the standard diet (AIN 93M); TP: a group received AIN 93M with tempeh flour; RS: a group received AIN 93M with soy; DG: a group received AIN 93M with pure daidzein and genistein; LA: a group received AIN 93M with *Lactobacillus acidophilus*; DGLA: a group received AIN 93M with daidzein, genistein, and *L. acidophilus*. ANOVA was performed using Tukey’s test. ^a–c^ represents significantly different mean values ± SD at *p* < 0.05.

**Table 4:** Percentage of myeloid and erythroid series in femoral bone.

Group	Myeloid Series (%)	Erythroid Series (%)	Ratio
K	61.25 ± 11.26	38.75 ± 11.26	1.83 ± 1.04
TP	63.75 ± 22.00	36.25 ± 22.00	2.57 ± 1.63
RS	71.25 ± 9.91	28.75 ± 9.91	2.79 ± 1.10
DG	67.50 ± 13.89	32.50 ± 13.89	2.63 ± 1.48
LA	63.75 ± 15.98	36.25 ± 15.98	2.31 ± 1.48
DGLA	68.75 ± 12.46	31.25 ± 12.46	2.63 ± 1.27

Data are presented as mean ± standard deviation. K: a group received the standard diet (AIN 93M); TP: a group received AIN 93M with tempeh flour; RS: a group received AIN 93M with soy; DG: a group received AIN 93M with pure daidzein and genistein; LA: a group received AIN 93M with *Lactobacillus acidophilus*; DGLA: a group received AIN 93M with daidzein, genistein, and *L. acidophilus*. ANOVA was performed using Tukey’s test with mean values ± SD at *p* < 0.05.

**Table 5:** Pearson’s correlation between the Mg concentration in serum and tissues in Wistar rats after the eight-week intervention.

	Liver—Brain	Serum—Heart	Serum—Hair	Kidney—Hair	Pancreas—Hair
*r*	0.299 *	0.320 *	−0.313 *	−0.280 *	0.548 **
*p*	0.026	0.018	0.021	0.031	0.000

The table displays the correlation coefficients (*r*) and significant values (*p*) for the relationship between Mg concentrations in serum and various tissues. The asterisk (*) denotes a significant *p* value below 0.05, while two asterisks (**) indicate a significant *p* value below 0.01. Only significant correlations are presented in the table, and correlations that did not reach statistical significance are omitted for clarity.

## Data Availability

The data presented in this study are available on request from the corresponding author.
